# HDAM: a resource of human disease associated mutations from next generation sequencing studies

**DOI:** 10.1186/1755-8794-6-S1-S16

**Published:** 2013-01-23

**Authors:** Meiwei Jia, Yanli Liu, Zhongchao Shen, Chen Zhao, Meixia Zhang, Zhenghui Yi, Chengping Wen, Youping Deng, Tieliu Shi

**Affiliations:** 1 Center for Bioinformatics and Computational Biology, Shanghai Key Laboratory of Regulatory Biology, the Institute of Biomedical Sciences and School of Life Science, East China Normal University, Shanghai 200241, China; 2 Department of Ophthalmology, West China Hospital, Sichuan University, Chengdu, Sichuan 610041, China; 3 Schizophrenia Program, Shanghai Mental Health Center, Shanghai Jiao Tong University School of Medicine, Shanghai 200030, China; 4 TCM Clinical Basis Institute, Zhejiang University of Chinese Medicine, Hangzhou, Zhejiang, 310053, China; 5 Rush University Cancer Center, Department of Internal Medicine, Rush University Medical Center, Chicago, IL 60612, USA

## Abstract

**Background:**

Next generation sequencing (NGS) technologies have greatly facilitated the rapid and economical detection of pathogenic mutations in human disorders. However, mutation descriptions are hard to be compared and integrated due to various reference sequences and annotation tools adopted in different articles as well as the nomenclature of diseases/traits.

**Description:**

The Human Disease Associated Mutation (HDAM) database is dedicated to collect, standardize and re-annotate mutations for human diseases discovered by NGS studies. In the current release, HDAM contains 1,114 mutations, located in 669 genes and associated with 125 human diseases through literature mining. All mutation records have uniform and unequivocal descriptions of sequence changes according to the Human Genome Sequence Variation Society (HGVS) nomenclature recommendations. Each entry displays comprehensive information, including mutation location in genome (hg18/hg19), gene functional annotation, protein domain annotation, susceptible diseases, the first literature report of the mutation and etc. Moreover, new mutation-disease relationships predicted by Bayesian network are also presented under each mutation.

**Conclusion:**

HDAM contains hundreds rigorously curated human mutations from NGS studies and was created to provide a comprehensive view of these mutations that confer susceptibility to the common disorders. HDAM can be freely accessed at http://www.megabionet.org/HDAM.

## Background

Mutation is a major cause of human diseases. Scientific studies of mutational mechanisms in human genes and the function of mutated genes in pathological pathway to disease are always research hotspots. With the success of next generation sequencing technologies in past several years, exome sequencing [1], target resequencing [2] and whole genome sequencing [3-5] applied in studying human disorders, such as monogenic diseases, complex inherited diseases and cancers, have already proven their powers to detect susceptible mutations. Thus, significant outcomes have been accumulated. Systems biology studies have shown its critical role in mechanism illustration and correlation prediction between mutation and disease/traits [6]. However, almost all of these studies have to take great extra efforts on data integration from publications for both mutation description standardization and disease category because of inconsistence between data descriptions from different resources. Inspired by the great achievement of some popular databases, such as the database of Genotypes and Phenotypes (dbGaP), which was developed to archive and distribute the results of studies that have investigated the correlation of genotype and phenotype [7], we believe that a database focusing on collecting mutation-disease relation and uniform mutation description will be meaningful and useful, especially with more and more data accumulated in future.

Nevertheless, mutations are frequently documented with only protein and/or DNA sequence without being clarified with reference sequence accession number or version, which make those mutations unavailable for re-analysis and integration. For example, mutation records in the Online Mendelian Inheritance in Man (OMIM) [8] Allelic Variant field often have such problems. Many early records are described as sequence variation at protein level only based on amino acid sequences reported in publication without protein accession IDs and versions [9]. Since the currently annotated protein often differs from these early sequences, the positions of some mutations cannot be inferred on the current sequences. In another word, such mutation records will lose their value in use.

To overcome these limitations, HDAM database is dedicated to collect and annotate mutations in nuclear genes underlying human diseases from published NGS studies. The core work is to uniform mutation nomenclature, locate mutation at different reference genome, record association between mutation and disease, and integrate meaningful annotation from both text-mined results and other databases. Particularly, the standard syntax of mutation description is critical for its sustainable utilization in both molecular effects and biological functions. Another major accomplishment of this article is to predict new mutation-disease association through combining OMIM Allelic Variants data. Mitochondrial genome mutations are not included in current release.

## Construction and content

### Literature mining

The mutation-disease relationship records in the current release were collected through PubMed searches using the terms '*(exome OR whole OR deep OR high-throughput OR (next AND generation) OR (massively AND parallel)) AND sequencing AND mutation'*. Through the abstract scanning, only the articles which focus on studying human diseases by the next generation sequencing were selected for further review.

Information on the following study-level fields was extracted from related papers: PubMed ID, PubMed URL, publication title, patient ID, patient ethnicity, the technique and the platform used in the study, disease information, validation experiments. Particularly, the disease name was checked by searching OMIM, Wikipedia (http://www.wikipedia.org/), Human phenotype Ontology (HPO) [10], Orphanet [11] and International Classification of Diseases (ICD, http://apps.who.int/classifications/apps/icd/icd10online/). If available, the OMIM IDs, HPO IDs and/or ICD numbers were also assigned to corresponding phenotype.

In the process of extracting mutation-level information, a crucial criterion has been followed: only the mutation that can be accurately located on genome is collected. The mutation-level information includes: Genomic position, base change on genome, transcript position, base change on transcript, protein position, amino acid change, gene, chromosome number.

Several problems were encountered during text mining: Firstly, not all above information can be extracted from every paper. For a mutation, if only position information on genome and base change are available, all possible cDNA level and protein level descriptions are kept. For example, mutation NC_000011.8:g.9794133G>A may be located either in gene LOC283104 or gene SBF2. Because of no more detail information about this mutation in original text, both of the two genes were collected into our database. Secondly, a gene may have several cDNA reference sequences. If the cDNA accession number for a mutation is not assigned, we preferred to keep all possible cDNA level descriptions that indicate the same amino acids mutation as shown in original articles. Besides, inconsistence between cDNA description and protein description was also observed in some articles. Under such circumstance, we used UCSC BLAT tool [12] to check and correct the discordance. During the whole data annotation process, we utilized Mutalyzer 2.0 β-8 [13] to check all collected data and complete information of each mutation when some information is not available from articles. Mutalyzer is a tool primarily designed to check descriptions of sequence variants according to the standard human sequence variant nomenclature of HGVS. Therefore, it makes it possible for us to standardize all mutation information from different articles. In this project, two independent reviewers extracted information from the same articles in parallel to ensure that they applied our inclusion criteria in the same manner. Then, all results were checked and corrected by a third reviewer.

### Data integration

To further help in understanding the relationship between mutation and disease, biological annotations under different levels were also integrated into HDAM. On the gene level, gene function annotation by Gene Ontology [14], OMIM gene description, sequence information was incorporated into the database. On the protein level, the domain annotation was accomplished by HMMSCAN (http://hmmer.janelia.org/search/hmmscan), and the protein structure 3D graph with mutation labels was visualized by Jmol.

OMIM represents the most complete and up-to-date repository of all known disease genes and the disorders. The "omim.txt" file was downloaded from OMIM website, consisting of 21,728 OMIM records. We parsed omim file and extracted gene records containing Allelic Variants (AV) field, which records the relations between the mutations or polymorphisms of the genes and the phenotypes. The disease-gene association mediated by mutations can be more proper for exploring pathological mechanism of mutation induced diseases. Totally, 16,574 mutations, 18,510 mutation-disease pairs and 4,549 gene-disease pairs were extracted from 2,604 gene records.

### The Bayesian network approach

As a representation of biofunctional system, Protein-Protein Interaction (PPI) network has been widely adopted in exploring the common or related mechanisms of complex phenotypes, such as network based function module discovery [6] and pathway tracing [16]. To facilitate understanding of the biological linkage between diseases and mutations, a Bayesian network based scoring system was built in our platform, which integrates both topological and biological features, including disease neighbor density by common module profiling score (CMP) [17], disease gene function similarity by Shared Smallest Biological Processes score (SSBP) [18]. A disease mutation preferential interaction score (DMPI), which represents the most frequently used PPIs that link the mutated genes in a given disease, was also featured in the Bayesian network. The DMPI is treated as an efficacious bridge that makes the level-2 neighbor in PPI network to be available in the prediction [19]. These three types of the features are hierarchically organized in the Bayesian network. Topological (CMP and DMPI) and biological features (SSBP) are conditionally independent, while CMP and DMPI are non-independent given a disease-mutation linkage. Golden Standard Positive (GSP) dataset contains the disease-mutation relations that were manually collected from published sequencing results and OMIM Allelic Variants. As less result is available for the non-disease oriented mutation, Golden Standard Negative (GSN) dataset was constructed by random combining diseases with the mutations that were annotated in a sufficient distance, where the distance was calculated in the HPO category tree. The confidence score for each inferred disease and mutation linkage was defined as the posterior probability of the hierarchical Bayesian network. Totally, 229,309 new mutation-disease relations have been predicted based on HPO, with Likelihood Ratio larger than 685 and FDR as 0.03.

### Record description

Each mutation recorded in HDAM was given a unique identifier. Each mutation has three level symbols, including genome, cDNA and protein. The symbol is constructed with sequence accession number in GenBank, sequence type symbol, mutation position and sequence change. For example, NM_020975.4:c.2944C>T refers a C→T substitution happened at position 2944 on cDNA sequence NM_020975.4; NP_009008.2:p.Arg42* indicates that Arginine at position 42 on protein sequence NP_009008.2 is mutated to a stop codon. All mutation symbols used in our database follow the HGVS nomenclature recommendations faithfully [20].

A HDAM entry comprises a mutation located gene, chromosomal location, protein domain, associated disease, the reference to the literature report of this mutation and some details in the literature (eg. The technique, platform, sample ethnicity and description of this mutation). Besides, annotation integrated from other sources, such as OMIM and GO, is also available. Moreover, predicted new mutation-disease pairs with Bayesian Network are presented in each mutation entry.

## Utility and discussion

### Utility

To provide a systemic gateway for mutation-disease relationship exploration, we implemented several search features on the website, including mutation description syntaxes (e.g. 'p.Gly1349Asp'), reported gene name (e.g. 'CFTR'), chromosomal region (e.g. 'chr1:100000-200000'), disease (string search or multiple option search), or OMIM disease IDs. To browse the results, the reference genome version (hg18/NCBI36 and hg19/NCBI37) must be selected before search. After submission, a result list is displayed, including mutation ID, mutation description on genome level, mutation description on cDNA level, mutation description on protein level, mutation type, mutation effect, associated diseases and PubMed IDs (Figure 1.1). To see more details about a mutation, the user can click the "view" button and a new page will appear to display all information about this mutation, both from manual collection and integration.

**Figure 1:**
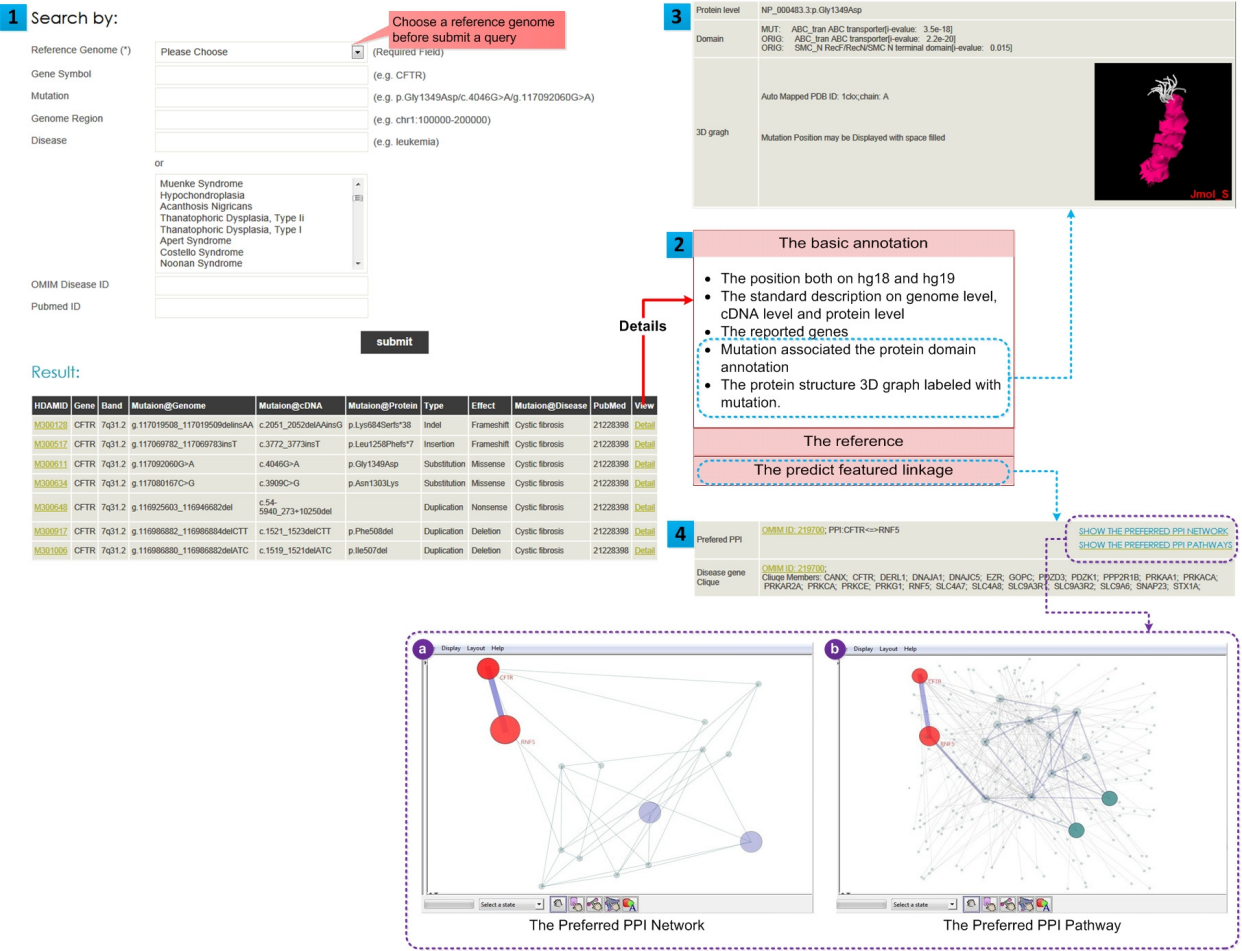
**Searching for information of a mutation in gene EGFR via the simple gene search interface**. The panel 1 on the upper left corner is the search interface of HDAM and result table. The panel 2 on the right in the middle of the figure illustrates the content of a mutation page. Panel 3 on the top right corner shows the protein domain containing mutation and the protein structure 3D graph labeled with mutation via Jmol. Panel 4 on the bottom right conner shows the preferred path in PPI network (a) and the preferred PPI network (b).

The mutation page can be separated into three sections: 1) the basic annotation, 2) the reference and 3) the prediction (Figure 1.2). The basic annotation includes: 1) the position both on hg18 and hg19; 2) the standard description on genome level, cDNA level and protein level; 3) the reported genes and the gene function annotation (GO); 4) the protein domain containing mutation and the protein structure 3D graph labeled with mutation (Figure 1.3). The reference information reveals the ethnicity report of the mutation, sequencing sample, methods, platform and description of mutation in articles. In the prediction results, new mutation-disease associations with scores and preferential proteins mediating such predicted linkages are shown to users. For example (Figure 1.4), the mutation NM_000492.3:c.350G>A (id: M301052)in gene CFTR is associated with Cystic fibrosis as reported with a significant preferred PPI with RNF5(PMID:21228398), which may contribute to new disease-mutation prediction in level-2 PPI neighbors. The preferred PPI network can be shown in GUESS applet in the link page "SHOW THE PREFERRED PPI PATHWAYS"(Figure 1.4.a), while, a smaller network with only pathway nodes contained in the link page "SHOW THE PREFERRED PPI NETWORK" (Figure 1.4.b).All neighbor genes will be listed in disease gene Clique tab if all the genes associated with the given disease are significantly enriched in the level-1 neighbors of the queried mutation

## Discussion

**Figure 2:**
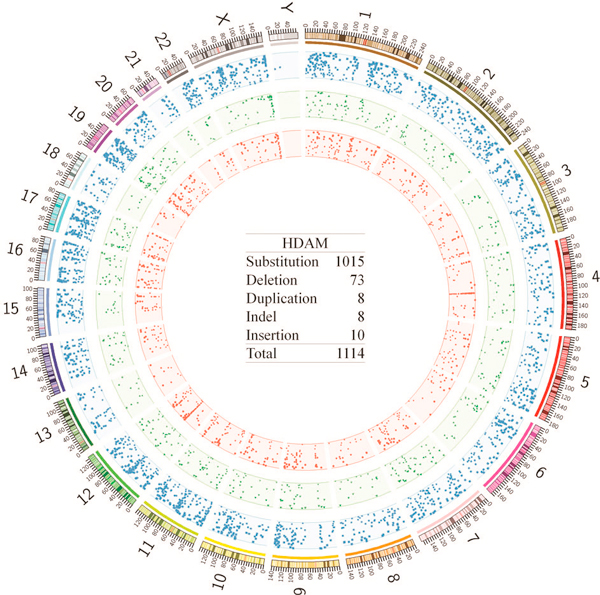
**Mutation and gene distribution on circos diagrams**. The outer circle represents the chromosomes of the reference genome hg19 with chromosome band labeled. The blue rectangles in light blue ring are the mutated genes in OMIM. The green triangles in light green ring are genes in HDAM. The red dots in light red ring are the mutations recorded in HDAM. The text in middle represents statistics of mutation types in HDAM.

HDAM currently contains 1,114 mutations, 669 genes and 125 human diseases (Figure 2). There are 444 genes in HDAM have no allelic variant records in OMIM. Actually, these genes discovered by NGS technology were confirmed with mutation events in patients, which have potential biological function and clinical applications. For example, a de novo missense mutation is identified in the gene TGM3 in an autistic proband [21]. TGM3 or Transglutaminase 3 belongs to transglutaminase family which are enzymes that catalyze the crosslinking of proteins by epsilon-gamma glutamyl lysine isopeptide bonds. In this protein family, another member TGM2 is related with autism spectrum disorders [22], indicating that there is a possibility that TGM3 has underlying effect on mechanism of autism. Therefore, new mutated genes discovered by NGS technology should be pointed out, in need of further validation experiments. The quality and quantity of data are the most essential and crucial characteristics for a database. Therefore, the mutation collection will be a long-term task with great efforts. Considering that mutation information is widely utilized in the biomedical researches, periodically updating genomic annotation of mutations (e.g. position) is necessary for several reasons: 1) novel mutations are published; 2) more annotation data and references of existent data need to be added into database; 3) new version of human reference genome is published; 4) bugs and comments are collected from feedbacks. To meet the demand, HDAM will be continuously improved and updated.

## Conclusion

HDAM contains hundreds of rigorously curated human mutations from NGS studies and was created to provide a comprehensive view of these mutations that confer susceptibility to the common disorders. These data will provide an available and standardized resource for large-scale studies. However, due to the unformatted mutation descriptions in the publications, it is a time-consuming and labor intensive task for the mutation examination and disease category. HDAM extends the effect of standardized database, and makes mutation descriptions more standard in publications. Large-scale mutation disease/traits studies provide more systematic and functional view by integrating amount of mutations, disease/traits, and also various types of omics data. As a core of genome-wide annotation, genomic position is considered as the most important criterion in data correction. Nonetheless, disease category is hard to be selected, updated and compared with others, which is more complicated than mutation correction. Thus, standardization in the related field is urgently in demand. HDAM tries to contain HPO, OMIM and ICD definitions simultaneously, and builds a mutation-directed mapping table for further evaluation. The new predicted mutation-disease relations based on integrating OMIM allelic variants data and protein-protein network also provide possible new underlying causes for the related diseases.

## Availability and requirements

The Human Disease Associated Mutation (HDAM) database is publicly available at http://www.megabionet.org/HDAM.

## Competing interests

The authors declare that they have no competing interests.

## Authors' contributions

TS and MJ conceived and designed the study. MJ, YL, ZS and CW collected the data, MJ and CZ performed the data analysis and built the system. TS, MJ and YD wrote the manuscript, TS finalize the manuscript.
